# The process of building European university alliances: a rhizomatic analysis of the European Universities Initiative

**DOI:** 10.1007/s10734-022-00898-6

**Published:** 2022-08-16

**Authors:** Antonin Charret, Maia Chankseliani

**Affiliations:** Department of Education, University of Oxford, 15 Norham Gardens, Oxford, OX26PY UK

**Keywords:** Higher education, University alliances, European Universities, European Universities Initiative, Rhizome, European Commission

## Abstract

Drawing upon French philosophy, this study offers a novel empirical and conceptual understanding of the newly launched European Universities Initiative. In 2019, higher education institutions across the European Union created 17 new alliances as part of the first pilot phase of the initiative. This is an experiment in European and global higher education. This paper offers a conceptual contribution to the field of higher education studies, making use of a rhizomatic analysis to explore how university alliances build what the European Commission refers to as the ‘European universities of the future.’ Based on the conceptual reflection and findings from a small-scale empirical study, this paper concludes that the alliances within the European Universities Initiative rely on pre-existing higher education and research partnerships while at the same time experimenting to foster a diversity of institutional forms to achieve the ambitious goal of creating ‘European Universities.’

## Introduction

Higher education institutions in the European Union formed 17 European university alliances in 2019. These new alliances are seen as stepping-stones for the establishment of ‘European Universities.’ The creation of these transnational alliances has been supported by the first pilot phase of the European Commission’s European Universities Initiative which aims to ‘enable students to obtain a degree by combining studies in several European countries and [to] contribute to the international standing and competitiveness of [European] universities’ (Von der Leyen, 2019). The idea of European university networks is not new. ‘Ideas do not arrive out of the blue’, explained Anne Corbett as she dated the origins of European university networks to the mid-1970s and examined the roots of the Bologna Process (Corbett, 2005, p. 8). The Bologna Process and later the Lisbon Strategy sought to increase the policy convergence of different national higher education systems. The European Universities Initiative builds on the Bologna Process and Lisbon strategy to develop ‘unprecedented levels of institutionalised cooperation, making it systemic, structural and sustainable’ (European Commission, 2020a, p. 131).

Higher education research has looked at the transnational partnerships of higher education institutions (HEIs) in Europe and globally. Vukasovic and Stensaker (2018) discuss university alliances in Europe as ‘interest groups’ and argue that the European University Association (EUA) or the League of European Research Universities (LERU) play an active role in policy making processes as they interact directly with EU institutions to defend the interests of their members. Chapman et al., (2014) look at cross-border university networks in Asia and Africa to show how these multi-university networks can strengthen the long-term social and educational agenda of participating universities. The advantages of cross-border networks come with the increase in the complexity of operations, concluded by Chapman et al., (2014). The ‘collaborative advantage’ of ‘strategic alliances’ of universities is also highlighted by Gunn and Mintrom (2013); they examine global university alliances such as the Association of Pacific Rim Universities, Universitas 21, and the Worldwide Universities Network. The four studies mentioned in this paragraph rely on similar empirical evidence in order to compare these transnational higher education partnerships: the year the alliance is founded, the  number of members, membership type, and geographical distribution.

This paper offers a new conceptualisation of multi-university networks. The purpose of the first pilot phase of the EUI is for the alliances to ‘test different innovative and structural models’ (European Commission, 2018b, p. 123). Each alliance is different. They vary in form, histories, and identities. This feature—difference—inspired the interest to comparatively examine these alliances through a rhizomatic framework. Comparative and international higher education scholarship encourages the development of new concepts that have the capacity to travel across national borders for the purpose of comparative research (see Rose & Mackenzie, 1991; Antonucci, 2013; Bleiklie, 2014; Seeber, 2020; Oleksiyenko et al., 2021).

**Fig. 1:**
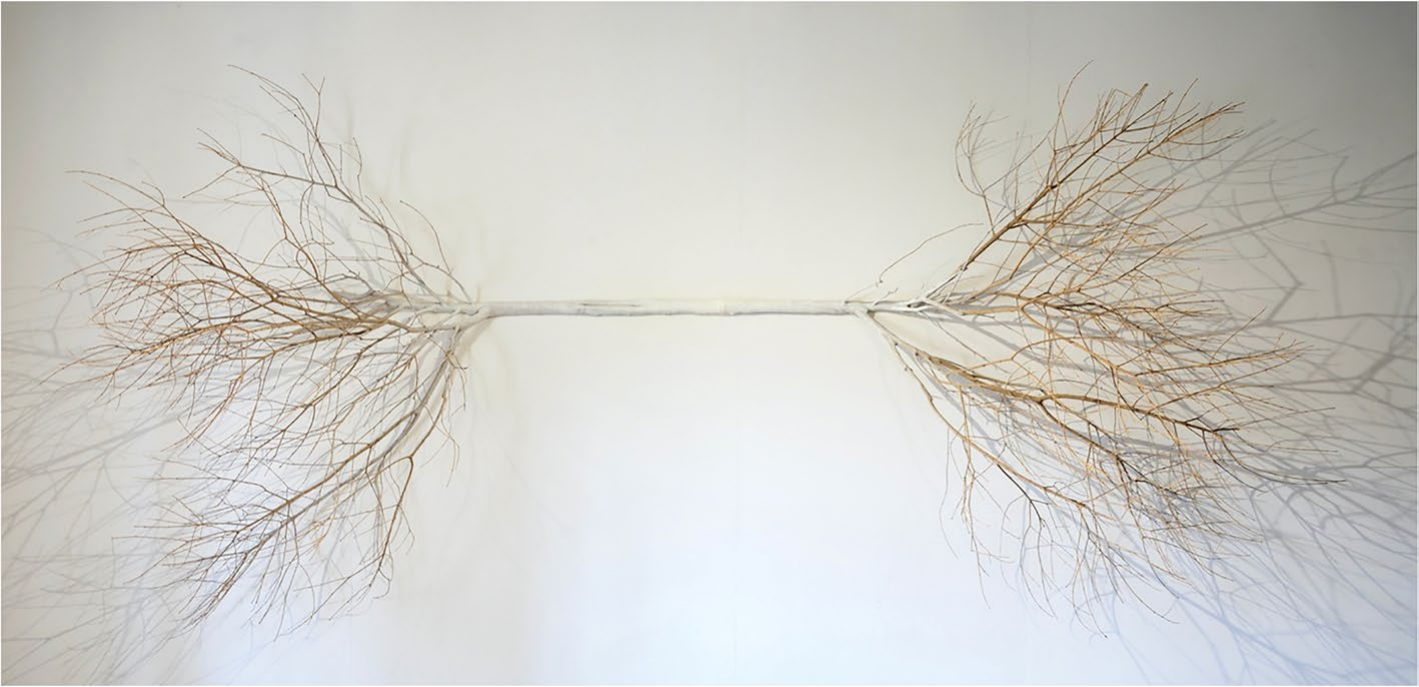
Cécile Beau, Rhizome, 2016, troène (Ligustrum Ovalifolium), dimensions variables. Source: courtesy of galerie 22,48 m2, Paris. Production: Le Parvis Centre D’Art Contemporain. Photo: Alain Alquier

**Fig. 2:**
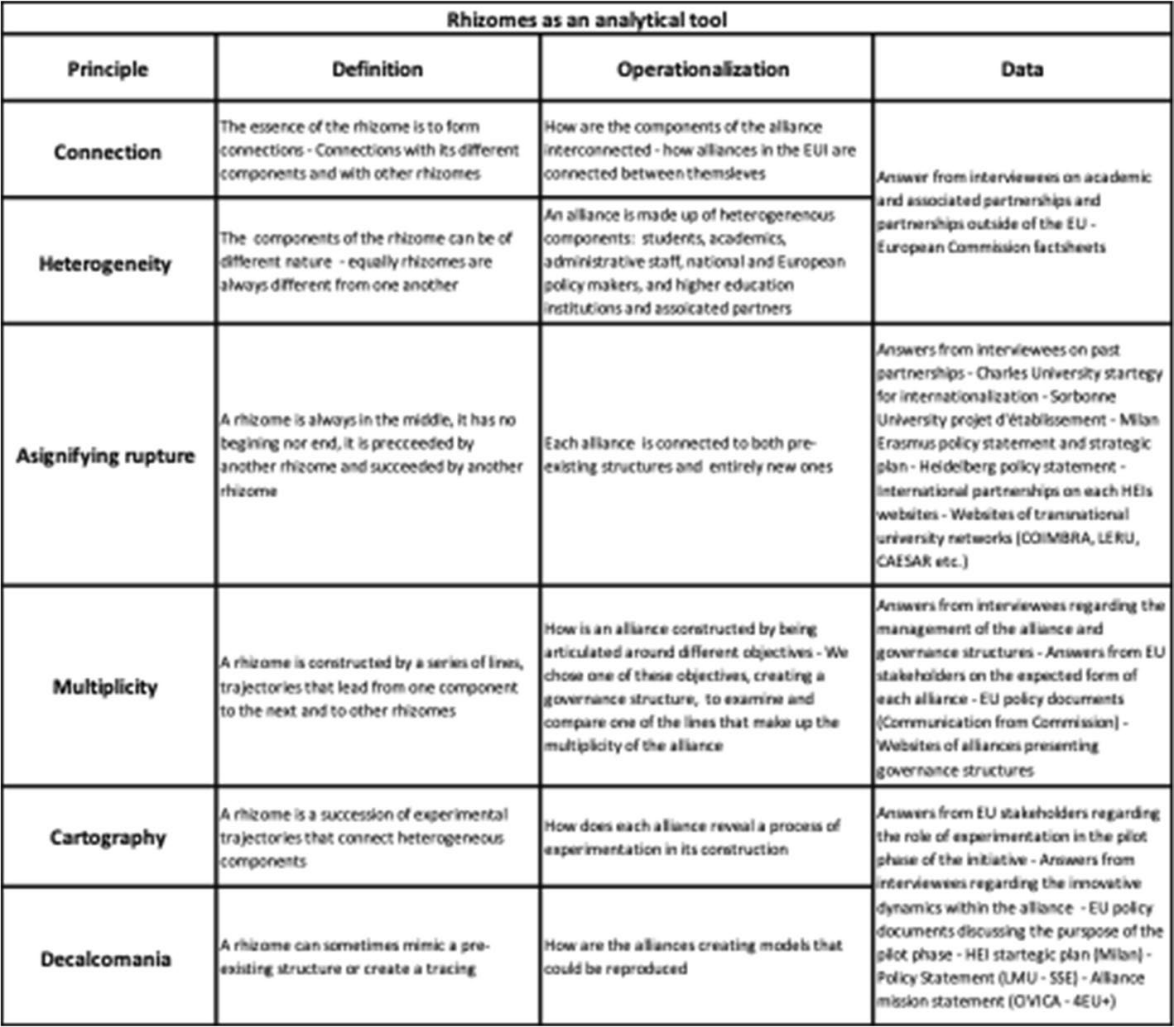
Operationalisation of the rhizome

A rhizome is found in the natural world. ​It is ‘the thick stem of some plants, such as iris and mint, that grows along or under the ground and has roots and stems growing from it’ (Oxford Learners Dictionary). In that sense, a rhizome’s growth is horizontal (Fig. 1) and differs radically from that of a root or a tree, which grow vertically and represent hierarchy. A rhizome has no easily identifiable central point. It flees hierarchy. Gilles Deleuze and Felix Guattari created this concept to be able to think about connections between heterogeneous entities. These entities do not derive from one main essence but are instead assembled from multiple origins that create a novel and unique whole. A rhizome reaches out in all directions and can exist in numerous ways. It draws lines and connects heterogeneous components together. It is a network made up of heterogeneous networks. In the introduction to *A Thousand Plateaus*, Deleuze and Guattari articulate the concept around six general principles, asignifying rupture, connection, heterogeneity, multiplicity, cartography, and decalcomania. This paper argues that each university alliance in the EUI can be seen as a rhizome that can be analysed and compared through these six principles (see Fig. 2). Therefore, this study moves beyond the criteria used by previous studies on transnational university alliances.

A rhizomatic analysis is different from a network analysis. The mainstream of network research is focused on the analysis of the nodes and edges of a network, measuring for example the sizes and degrees of nodes, or finding patterns in the relationship between them. A rhizomatic analysis can also examine such characteristics through the principles of connection and heterogeneity. However, in a rhizome, each component is affected by one another in a contagious manner. Crossley (2010) argues that social network analysis does not give the possibility to investigate the meanings or the reasons for interactions within networks. Unlike a network structure which is only ‘defined by a set of points and positions, with binary relations between the points and biunivocal relationships between the positions, the rhizome is made only of lines’ (Deleuze & Guattari, 1988, p.21). These lines are what Deleuze and Guattari call multiplicities. These multiplicities, or lines, are the different routes the rhizome takes to accomplish itself. Deleuze and Guattari are specifically interested in how these different lines (or multiplicities) construct a cartography that make up a rhizome. A rhizomatic analysis facilitates the study of multiplicities and the cartographies that rhizomes build.

Deleuze and Guattari consider that the point is never to ask what a rhizome means or what it is but rather, how it functions. This has guided the overarching research question that this paper addresses: How are the university alliances of the European Universities Initiative formed and how do they function in practice? At the forefront of this investigation is the nature of the interaction between various components of an alliance and the ways in which they draw lines to build a cartography—a rhizome. We explain what the components of an alliance are in the next section; however, this paper is less concerned with the nature of the components found in an EUI alliance, in other words the nodes and edges for which a social network analysis might have been useful.

The analysis of the university alliances of the EUI in this paper is guided by the six principles of the rhizome to address the following sub-questions: Where do these novel university alliances come from (asignifying rupture)? What are these alliances composed of and through which connections do they function (connection and heterogeneity)? How diverse are these alliances in their objectives (multiplicity)? How do they lead experimentations while also being constrained by pre-determined objectives (cartography and decalcomania)?

This study uses rhizomes as an innovative comparative framework (see Fig. 2). The choice of the research design was inspired by a preliminary impression that the concept from philosophy—rhizomes—could be helpful in exploring the emergence of European Universities. The principles of connection and heterogeneity give an insight into the creation of a higher education network built on the interconnected components of different nature. The principle of asignifying rupture offers a lens with which to witness how the alliances of the EUI build on previous transnational networks while creating new ones. Multiplicity is used to see how the diversity of the alliances is displayed through different objectives or approaches to attain common goals. The principle of cartography gives the possibility to discuss the intention of alliances to experiment to find solutions towards closer transnational cooperation. Decalcomania, or the mania of calques (tracings), imposes a pre-determined structure onto an object. Although the cartography is experimental, creative, and innovative, it is at times influenced by pre-existing structures. Experimentations are encouraged both in the rhizome and the European Universities Initiative. At the same time, tracings are not entirely absent.

The following section outlines the operationalisation of the six principles of the rhizome and the data used to explore the alliances of the European Universities Initiative. We then detail our research design and methods before presenting our findings that are set out in four following sections corresponding to the six rhizomatic principles: (1) asignifying rupture, (2) connection and heterogeneity, (3) multiplicity, and (4) cartography and decalcomania.

### Connection and heterogeneity: a rhizome’s components and how they fit together

Deleuze and Guattari present the rhizome as an ‘alliance, uniquely alliance (…) the fabric of the rhizome is the conjunction, “and... and... and...”’ (Deleuze & Guattari, 1988, p. 25). A rhizome can be composed of an infinite number of components that are meshed together without relying on any sense of hierarchy. What counts is to continuously connect one point to the next. It perpetually draws new connections, collaborations, and partnerships. A rhizome can be composed of people, institutions, concepts, or physical spaces. The emergence of the notion of alliances to define rhizomes resonated with the current formation of these European university alliances. An alliance is composed of heterogeneous components such as students, academics, higher education institutions, EU institutions, national governments in virtual and physical spaces. It is the interaction between these heterogeneous components that makes up an EUI alliance. Each alliance is also different and interconnected.

This study initially identified heterogeneous components of alliances through the factsheets published by the European Commission. These factsheets were used in preparation for interviews as they offered an overview of the different higher education institutions and associated partners participating in the alliances. Participants were asked questions about the nature of the interaction between various partners. The interviews also explored the connections between different alliances.

### Asignifying rupture: a rhizome’s origin(s) … and future(s)

The principle of ‘asignifiying rupture’, refers to how the rhizome is capable of being ‘broken, shattered at a given spot, but it will start up again on one of its old lines, or on new lines. (…) These lines always tie back to one another’ (Deleuze & Guattari, 1988, p. 25). Deleuze and Guattari define the rhizome as having ‘no beginning or end; it is always in the middle, between things, interbeing, intermezzo’(Deleuze & Guattari, 1988, p. 25). Being situated in the middle, the lines and dimensions found within the rhizome come from previous structures and form new ones. The alliances formed as part of the European Universities Initiative are precisely in the middle. The alliances are founded on previous partnerships and collaborations between the higher education institutions involved and precede the formation of novel networks that will be the intended European Universities. Thus, the pilot phases of alliances are ‘in the middle’, building on their existing partnerships in Europe and aspiring to be part of new transnational institutions.

To identify past partnerships, this study initially relied upon the interview evidence on the history of each alliance, with a particular focus on how participating universities joined forces. These initial responses led to further investigation of past connections between institutions making use of University strategy papers on internationalisation, Erasmus policy statements, and the information on universities’ international partnerships from their websites. As the role of past networks became apparent, this study also examined the websites and position papers of transnational university networks, such as COIMBRA, LERU, UNICA, SGroup, GPPN, APSIA, and CAESAR.

### Multiplicity: a rhizome can develop in a variety of ways

A rhizome is made up of multiplicities. Multiplicities are defined as lines rather than points (Deleuze & Guattari, 1988). This is perhaps the key characteristic that differentiates a rhizome from a traditional understanding of networks made up of a series of nodes and edges. The lines of a rhizome should not be seen as an edge connecting node A to node B. Multiplicities should be seen as the routes taken by all of the components found within a rhizome. A network analysis looks at the direction of the edges, understanding the direction of the flow that operates between two points. A rhizomatic analysis offers the possibility to explore the trajectories being used and how they function. De Landa (2002) presents multiplicities as the ‘structure of spaces of possibilities’ (De Landa, 2002, p.3). The principle of multiplicity is used here to explore the EUI as the ‘space of possibilities’ through which each alliance can choose to unfold. Each singular choice and objective set by an alliance creates a trajectory between its components. The multiplicity of the rhizome is precisely these lines that draw all of the different paths that can be taken by the alliances.

The most suitable analysis of the multiplicities of rhizome would require the analysis of the processes of experimentation through which the alliances are built. The data for this study was collected at the start of the initiative. Hence, the study presents lines that have been put in motion. At that stage, only projected lines rather than the actual lines could be observed. During interviews, participants were asked about the form of their alliance: their objectives, their governance structures and the partnerships being drawn. The interview data helped to identify the documents and web-pages overviewing these objectives and governance structures. The interview data and document analysis revealed differences between alliances in the way they participate in the European Universities Initiative and the different institutional forms they each imagine for a European University. Although all of these rhizomatic lines were discussed in interviews, this paper focuses on the analysis of different governance structures found in the alliances.

### Cartography and decalcomania: a rhizome’s capacity to both experiment and mimic

The cartography of the rhizome is made up of all four components discussed above. The rhizome is a ‘map’ in which many itineraries, or multiplicities, are possible to connect heterogeneous components (Deleuze & Guattari, 1988, p. 12). Furthermore, the rhizome is not a finite cartography, it is situated ‘in the middle’ of a pre-existing cartography and an entirely new one in the making. Antonioli (2010) argues that the practice of cartography is led through imagination and the imaginary. She draws on Tiberghien (2007) who uses the example of Columbus producing a map imagining new routes to India prior to embarking on his journey. By imagining these new routes, Columbus is imagining the world. This representation constructs an image which is in no way a mimesis… a tracing. A cartography is not a ‘representation but a tool for experimentation and creation’ (Antonioli, 2010, p. 4). The rhizome offers the possibility to ‘think’ a network prior to its actual existence.

The map is therefore something which needs to be constructed. It is a surface upon which all the different heterogeneous components of the rhizome can create their connections, express their multiplicities, and display their origin. The map is the surface upon which to ‘think’ these novel university alliances. This surface is fresh and does not mimic anything else. A mimicry would correspond to a tracing. A rhizome is different to ‘the graphic arts, drawing, or photography, unlike tracings, the rhizome pertains to a map that must be produced, constructed, a map that is always detachable, connectable, reversible, modifiable, and has multiple entryways’ (Deleuze & Guattari, 1988, p. 21). The pilot phases of the European Universities Initiative function like a cartography with the alliances in a dynamic of experimentation.

Despite the cartographic structure for the European University Initiative, the European Commission (2018b) also reveals the importance of creating alliances that will serve as models, or tracings, for future alliances. The principle of decalcomania is introduced by Deleuze and Guattari as the final principle of the rhizome. Although the authors strongly advocate for maps to be built, they admit that rhizomes might contain some elements of tracings, with ‘knots of arborescence in rhizomes, and rhizomatic offshoots in roots’ (Deleuze & Guattari, 1988, p. 20). Cartographies and tracings are not completely opposite and can exist in a state of complementarity. The tracings which are present in the European Universities Initiative do not reject the principle of cartography. Instead, the practices of cartography lead to tracings, as this paper demonstrates. The experiments led as part of the pilot phases may eventually be used to create models for future alliances.

The data collected in this study only reflects one point in time. It is, therefore, not possible to analyse a process through which the cartography of an alliance is being constructed. However, the fact that the data was collected very shortly after the launch of the alliances offered an opportunity to learn first-hand about the purpose of the pilot phase, which revealed an intention to experiment and create models—cartographies and tracings. This study also looked for the presence of these intentions in the relevant EU documents on the goals of the pilot phase, as well as Erasmus policy statements and university strategy papers discussing the objectives of the pilot phase.

## Methodological approach

Semi-structured interviews and document analysis were the two key sources of evidence used in this study. The document analysis included communications from the European Commission, conclusions and resolutions from the Council of the European Union and European Council, the mission statements of the alliances, and at HEI level, documents relating to international and European strategy as well as Erasmus policy statements (Annex 1). The document analysis informed the understanding of the policy, legal, and institutional frameworks guiding the European Universities Initiative. The documents analysed shed light on the objectives of each alliance, the strategic priorities of participating HEIs and their previous partnerships.

The semi-structured interviews were used to explore interviewees’ experiences in relation to the formal institutional declarations selected from the document analysis. Thus, interviews built on the evidence from the document analysis. At the same time, interviewees helped to identify other relevant documents which were included in the document analysis. Three European university alliances were selected for semi-structured interviews. These three alliances bring together 18 higher education institutions from 10 EU member states (Table 1).

**Table 1:** Participants of the study: alliances and their constituent higher education institutions

Alliance	Constituent higher education institutions
CIVICA-The European University in social sciences	European University Institute (Italy) Handelshogskolan i Stockholm (Sweden) Hertie School (Germany) Institut d'Études Politiques de Paris (France) Kozep-Europai Egyetem (Austria and Hungary) Scoala Nationala de Studii Politice si Administrative (Romania) Universita Commerciale Luigi Bocconi (Italy)
EUGLOH-European University Alliance for Global Health	Université Paris-Saclay (France) Ludwig-Maximilians-Universität Muenchen (Germany) Lunds Universitet (Sweden) Szegedi Tudomanyegyetem (Hungary) Universidade do Porto (Portugal)
The 4EU + Alliance	Københavns Universitet (Denmark) Ruprecht-Karls-Universität Heidelberg (Germany) Sorbonne Université (France) Universita Degli Studi Di Milano (Italy) Univerzita Karlova (Czech Republic) Uniwersytet Warszawski (Poland)

All three alliances are coordinated by Paris-based HEIs. However, they differ with respect to the following characteristics: the identity and themes/disciplinary focus of each alliance, the nature and size of the HEIs making up the alliance, their past links with alliance members, and their governance structure.

CIVICA has an objective of becoming the European University of social sciences. As such, the participating higher education institutions all specialised in social sciences. The EUGLOH alliance brings together larger institutions than CIVICA, including excellence clusters such as Ludwig-Maximilians-Universitaet Muenchen and Paris-Saclay which encompass a variety of disciplines and serve large numbers of students. The higher education institutions that are part of this alliance serve 210,000 students, whereas an alliance such as CIVICA serves only 50,000 students in total.

The 4EU + alliance is an example of an early European university alliance. The 4EU alliance was established in March 2018 when the University of Warsaw, Charles University, Sorbonne University, and Heidelberg University formally established a European university alliance (University of Warsaw, 2018). The establishment of this alliance was preceded by the Communication of the European Commission at the end of 2017 on ‘Strengthening European Identity through Education and Culture.’ Thus, the alliance originates from the period prior to the publication of the European Commission’s ‘European Universities’ call in 2018.

The semi-structured interviews were conducted with the coordinators of these three university alliances, as well as project managers in charge of the European Universities programme at the higher education institutions that are part of the alliance, and representatives of the European Commission who coordinate the initiative. Thus, the study aimed to analyse every layer of the alliance in order to explore network processes. All interviews were conducted online by the lead author.

Overall, nine interviews were conducted. Two interviews in the 4EU + alliance (programme managers in charge of the European Universities programme in two partner higher education institutions), three interviews for the CIVICA alliance (the coordinators of the alliance and programme managers in charge of the European Universities programme in two of the higher education institutions), three interviews for the EUGLOH alliance (the coordinators of the alliance and programme managers in charge of the European Universities programme in two of the higher education institutions), and one interview at the Directorate General of Education and Culture of the European Commission. The interviews explore the ways in which these alliances were built, including governance structures, and the relationship between the alliances and the European Commission.

Interviews were conducted in Spring 2020. At that time, these alliances were very young. Some of the projects that had been planned, such as the inclusivity of students, or partnerships with the private and third sectors, were still at an early stage, if developed at all. The COVID-19 crisis evidently played a role in this. The alliances were all launched in autumn 2019, coordinators were mostly hired in January or February 2020, and the first measures of pandemic-related lockdowns were put in place in March. Nevertheless, the crisis also accelerated certain aspects of the universities, such as the development of tools for institutional collaborations or an unavoidable expansion of distance learning. This study underestimated the importance of academics and students in understanding the formation and functioning of these alliances. Future studies exploring the alliances will need to include the perspectives of academics and students.

A two-stage coding procedure was used for the analysis of the document and interview data. The first stage of the analysis involved coding based on the six principles of the rhizome: asignifying rupture, connection, heterogeneity, multiplicity, cartography, and decalcomania. This was followed by the second stage of analysis which involved the coding of emerging findings not directly linked to the conceptual framework. The latter was entirely exploratory. Subsequently, relationships were established between these two types of codes to produce a dialogue between them. The findings reported below build on the relationships between these two types of codes and try to create a correspondence.

## Asignifying rupture: building on past networks and partnerships

**Map 1:**
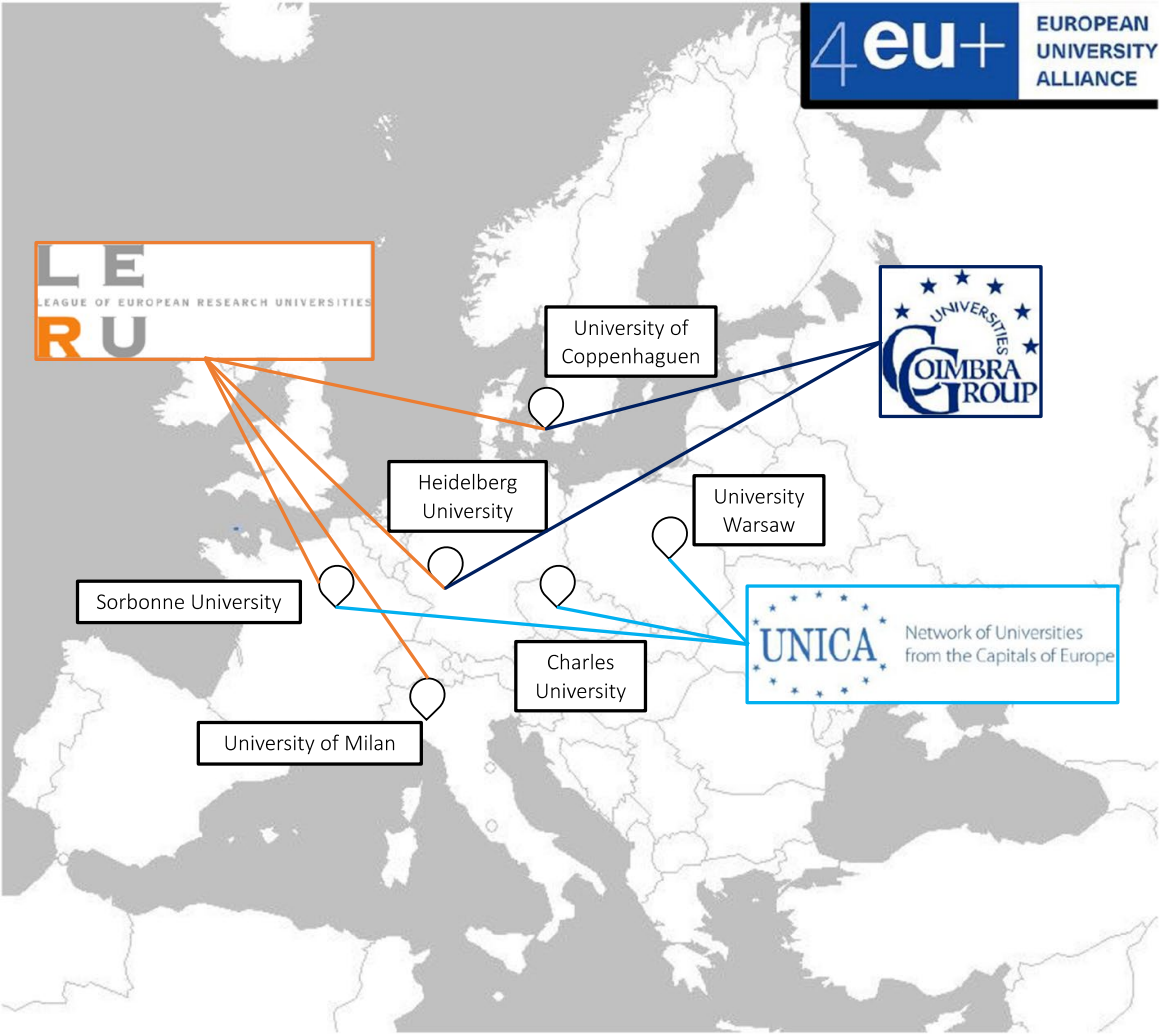
4EU + Alliance: Membership in Transnational University Networks. Source: this map was generated based on the information from our interviews, Charles University (2018), Sorbonne Université (2019), Heidelberg University (2020), University of Milan (2020a), University of Milan (2020b)

Rhizomes stem from a variety of origins. This study reveals that some European university alliances originate from previous alliances which existed as research networks. One of the participants of the 4EU + alliance discussed their presence in the ‘League of European Research Universities (LERU) which might have been the initial motif of putting [the original] 4 universities together’. He went on to state that they were ‘very active in the COIMBRA group (…). So coincidently, most of the member universities, have already been or are currently involved in other European universities projects.’ Thus, in the case of 4EU + , the four founder HEIs were part of the LERU research network (Map 1). This created a trust-based foundation for the partnership. The presence of LERU is something recurrent in the international strategy documents of 4EU + member HEIs (Charles University, 2018; Sorbonne Université, 2019; University of Milan, 2020a, 2020b). The analysis of the Erasmus policy statements and the HEIs’ websites also revealed the importance of being members of  the COIMBRA group and the UNICA network (Heidelberg University, 2020).

**Map 2:**
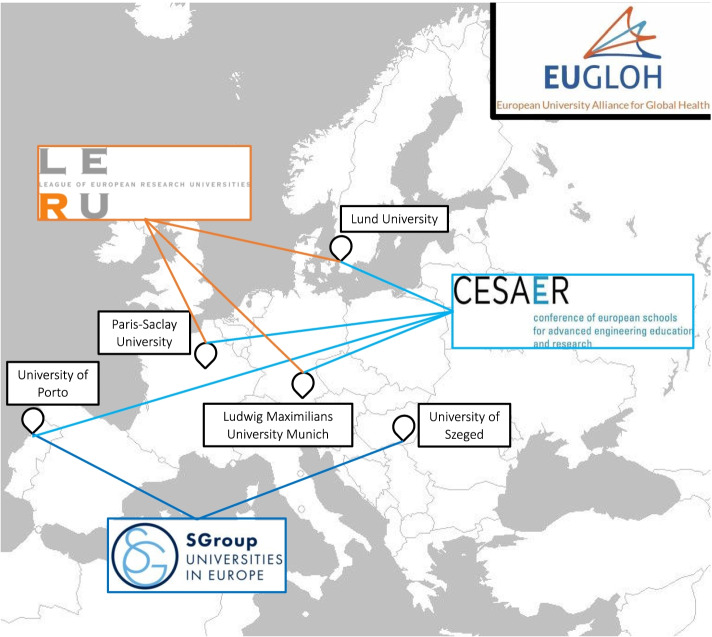
EUGLOH Alliance: Membership in Transnational University Networks. Source: this map was generated based on the information from our interviews, Ludwig Maximilian University of Munich (2020), University of Szeged (2020), CESAER (2021), LERU (2021), SGroup (2021)

A similar dynamic can be seen in the EUGLOH alliance (Map 2). One of the participants from EUGLOH explained that their institution was ‘in several networks, LERU, for example or CAESAR (…). Three of the universities were part of LERU. Four were part of CAESAR’. She went on to give an example of one of the partner institutions for whom it was important to develop a partnership with institutions that were part of LERU. They were not originally interested in being part of the alliance until they ‘saw that other [national] universities were getting involved, so they decided not to stay alone in their corner. They were right to do so, because all of the Universities of LERU are now in alliances.’ Beyond LERU and CESAER, an analysis of the international strategy and past partnerships of members of the alliance revealed the participation in the Sgroup (formally known as Santander Group of Universities).

**Map 3:**
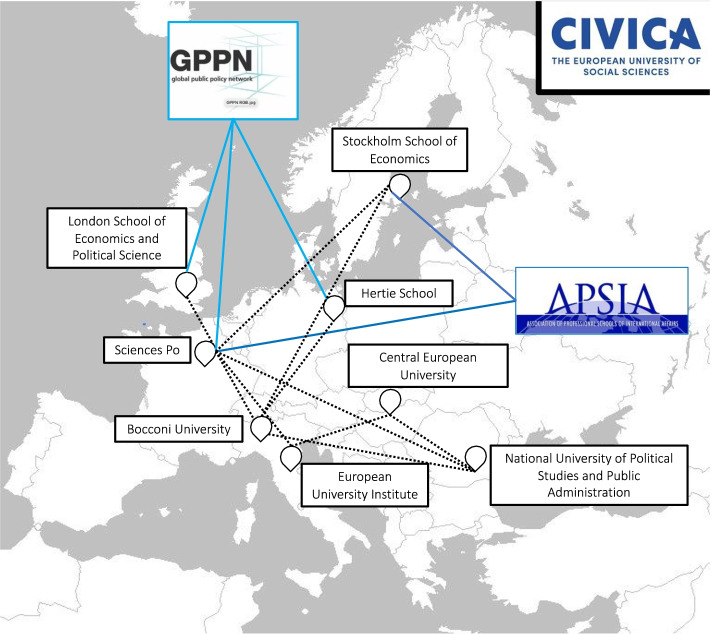
CIVICA Alliance: University-to-university Partnerships and Membership in Transnational University Networks. Source: this map was generated based on the information from our interviews, APSIA (2021), Bocconi (2021), European University Institute (2021), GPPN (2021), Hertie School (2021), Sciences Po (2021), SNSPA (2021), Stockholm School of Economics (2021)

In contrast to 4EU + and EUGLOH, the membership of these transnational higher education organisations was not highlighted by interviewees from CIVICA and did not feature in the alliance’s mission statement. Our study of the HEIs’ webpages did reveal common membership in transnational higher education organisations such as the Global Public Policy Network (GPPN) and the Association of Professional Schools of International Affairs (APSIA). However, the interviews and EUI-related documents emphasised the importance of previous collaborations with various higher education institutions across Europe to offer joint degrees, exchange programmes, or staff exchanges (Map 3). A participant from CIVICA explained that Science Po and the European University Institute were linked through the president of the European University Institute, Renaud Dehousse, who was the founder and former director of the Centre for European Studies at Science Po. The analysis of the international strategy documents of the HEIs that constitute CIVICA revealed numerous university-to-university partnerships that fostered exchange programmes and joint-degrees. Other collaborations included partnerships through Erasmus + which is the case of SNSPA with Sciences Po, Bocconi University, and the Central European University.

## Connection and heterogeneity: ‘pioneers’ and ‘associates’

Rhizomes are a series of connections of heterogeneous elements. The network of a university alliance very much reflects that of any single higher education institution. They are made up of heterogeneous components such as students, academics, administrative staff, university buildings, links with local, national, or regional public, and private stakeholders. Shortly after the results of the first EUI call, the European Commission published factsheets on each of the alliances. Each factsheet offers a glimpse into the heterogeneous networks that were to be formed. These networks were split in two: on the one hand the internal network formed by the participating higher education institutions, dubbed ‘the pioneers’, and on the other the external network that contributed to the development of the alliance, labelled ‘the associates’ (European Commission, 2020c, 2020d, 2020e). Interviewees at DG EAC also presented the alliances ‘as pioneers and role models for the other [European HEIs].’

The 4EU + and EUGLOH factsheets both display a comparable number of associates: 23 and 30, respectively. 4EU + displays strong ties with public institutions such as municipalities, regional authorities, and chambers of commerce. The links with the private sector are notable; out of the 23 associates, 12 are enterprises (Table 2). EUGLOH too has links with the private sector, but only 6 out of 30 associate partners are enterprises. This alliance has a more diverse list of partners than 4EU + but they are presented as broad categories within the network: clusters/PPPs, large-scale infrastructures, and research centres (Table 3).

**Table 2:** 4EU + : pioneers and associates.

Alliance	6 pioneers	23 associates
The 4EU + Alliance	Københavns Universitet (Denmark) Ruprecht-Karls-Universität Heidelberg (Germany) Sorbonne Université (France) Universita Degli Studi Di Milano (Italy) Univerzita Karlova (Czech Republic) Uniwersytet Warszawski (Poland)	12 Enterprises 4 Chambers of Commerce 3 NGOs 3 Municipalities 1 Regional Authority

Source: European Commission (2020c).

**Table 3:** EUGLOH: pioneers and associates

Alliance	5 pioneers	30 associates
EUGLOH–European University Alliance for Global Health	Université Paris-Saclay (France) Ludwig-Maximilians-Universität Muenchen (Germany) Lunds Universitet (Sweden) Szegedi Tudomanyegyetem (Hungary) Universidade do Porto (Portugal)	6 Enterprises 5 Clusters/Public Private Partnerships 1 Technology Transfer Office 2 regional authorities 1 NGO/Association 11 Research Centres 4 Large-Scale Infrastructures

Source: European Commission (2020e).

Of the three alliances examined in this paper, EUGLOH had the most extensive network of associates. The link with the private sector was seen as essential to meeting student needs: ‘we are speaking of students, training, and therefore competences, it means speaking of potential recruiters.’ The same participant shared how they started their relationship with two large-scale companies; they asked the company representatives: ‘Are you interested to have people tomorrow, including students completing professional bachelor’s degrees? Are you interested in becoming more European?’ The companies saw an advantage in supporting students to become ‘more autonomous, and having a better understanding of what Europe is.’ Another interviewee from the EUGLOH alliance justified the importance of building links with the world outside higher education to achieve the alliance’s mission pertaining to lifelong learning. With health as a central theme for EUGLOH, the participant highlighted how important it was to ‘educate nurses, and people that can on a short notice, enter hospitals, and work there because of the Covid crisis.’ The interviewee called this their ‘third task’, after research and education. This third task was dedicated to ‘learning outside of the universities.’

CIVICA is the only alliance from the first pilot call that chose another higher education institution—LSE—as an associate (Table 4). LSE was involved in the application process from the very start. Interviewees explained that keeping LSE as an associate rather than a pioneer was linked to Brexit. When asked why the current factsheet did not present any associates besides LSE, participants from the CIVICA alliance highlighted this as a realist choice; it reflected the difficulty of choosing and associating with non-HE partners.

**Table 4:** CIVICA: pioneers and associates (European Commission, 2020d)

Alliance	7 Pioneers	1 Associates
CIVICA-The European University in social sciences	European University Institute (Italy) Handelshogskolan i Stockholm (Sweden) Hertie School (Germany) Institut d'Études Politiques de Paris (France) Kozep-Europai Egyetem (Austria and Hungary) Scoala Nationala de Studii Politice si Administrative (Romania) Universita Commerciale Luigi Bocconi (Italy)	1 Higher Education Institution: London School of Economics and Political Science (UK)

One of the interviewees stated that when the partners first got together they told themselves: ‘let’s first focus on ourselves as HEIs because we know each other, we understand each other, we all have similar outlooks and similar questions in this endeavour.’ The same participant explained that it was ‘hard enough to coordinate between 8. Bringing in non-HE partners and seeing whether we make them associates and what role we would give them at that stage seemed to be early.’ Much like CIVICA, the ‘ARQUS’, ‘EPICUR’, and ‘UNITE!’ alliances, all made a similar choice: they have no associates listed on their factsheets.

Although the factsheets do not demonstrate any non-HE links, participants from CIVICA gave us a glimpse into what these links could look like. The participant considered that ‘actors that should be involved with HE should be businesses [and] members of local authorities, that could be city councils but also government levels, ministry levels, people that are actually involved in the policy making. They are indirectly also a target group, but also partners of this.’

Despite the long list of associates present in the factsheet, at the time of the interview, 4EU + interviewees mentioned that the different roles that the associates could have were merely ‘ideas.’

## Multiplicity: diversity of the alliances’ governance

**Fig. 3:**
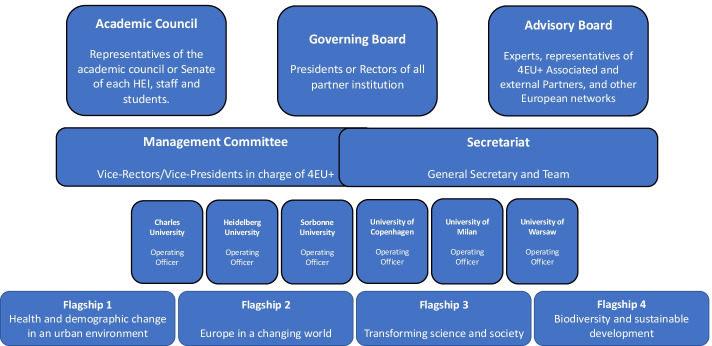
4EU+ Governance. Source: this figure was generated based on the information from our interviews and (4EU+, 2021)

**Fig. 4:**
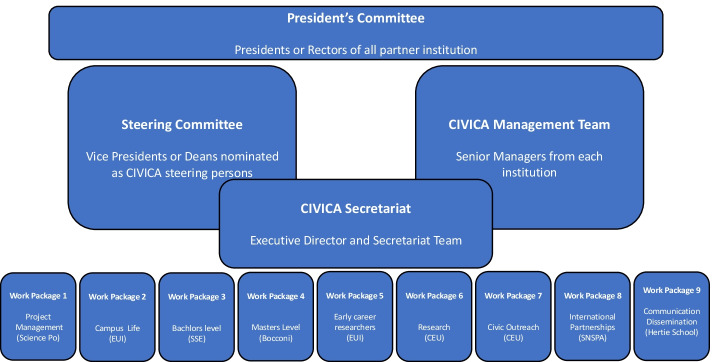
CIVICA Governance structure. Source: this figure was generated based on the information from our interviews and (CIVICA, 2021)

**Fig. 5:**
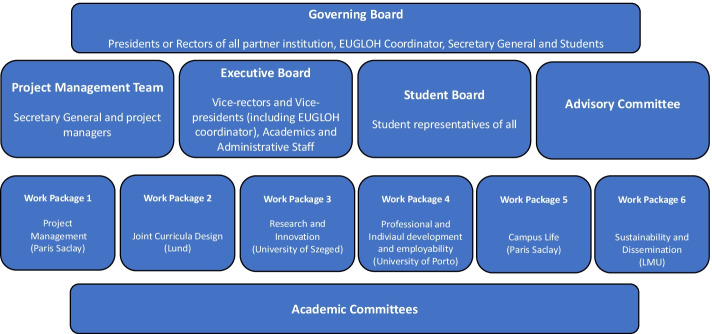
EUGLOH governance structure. Source: this figure was generated based on the information from our interviews and (EUGLOH, 2021)

The three alliances are all diverse in their origin, in the internal and external networks they develop, and also in their strategies to become the European universities of the future. Different strategies adopted by the alliances highlight their diversity and establish their multiplicities. The Commission indicated in the original communication about the initiative that there would ‘be no one-size-fits-all model. Institutions can propose the model that suits their needs through a bottom-up, open and transparent approach and develop the level of ambition gradually’ (European Commission, 2018a). Interviewees from DG EAC revealed that this was an incentive to promote the diversity of these alliances reflecting the diversity of the HE landscape within the European Union. Although all alliances share this goal of embodying these ‘universities of the future’ in the European Union, they do not necessarily have the same idea of what this means for the organisation of their governance structures. In Figs. 3, 4 and 5 below the paper presents three different models of governance structures based on the three alliance’s websites and information that was shared in interviews.

All three alliances examined in this study have a top governance level, which embodies the alliance’s strategic and political dimension. It is where the rectors or presidents of each participating institution meet. The three alliances have all hired a main coordinator to lead the alliance within the management team of the governance structure. 4EU + and CIVICA created an independent secretariat led by this coordinator, which exists alongside the management Team or Committee (see Figs. 3 and 4). In EUGLOH, the secretary general is fully embedded within the project management team (see Fig. 5).

One of the participants from the CIVICA alliance explained that the alliance’s governance very much relied on consensus, ‘it is not the type of alliance where you have the leader that acts as the manager and says you need to do this and that.’ Agreements are reached through discussion which ensures that each partner feels ownership of and responsibility for decisions. In contrast, one participant from 4EU + presented the governance model as entirely top-down, with the governing board, followed by the management committee coupled with the secretariat, and finally the project officers below (see Fig. 3). The participant presented the secretary general as the central component of the governance structure around which the rest is organised. Above the Secretary General is the Governing Board that sets the policies of the alliance and develops the strategic orientations. Below, the management committee is intended to be more operational. In CIVICA, a management team exists alongside a steering committee (see Fig. 4). One of the interviewees explained that the steering committee made operational decisions.

The involvement of students in governance emerged as one of the key differences between the governance structures of the three alliances. While discussing the potential involvement of students, a participant from CIVICA highlighted the difference between ‘governance’ and ‘associating different communities.’ The alliance sought to associate students and academic staff linked to different activities. However, when starting to develop the alliance, they did not want a ‘board of 40 people, complicated to govern.’ In a second phase of its construction, CIVICA planned to set up an academic committee and a student committee.

A participant from the 4EU + alliance similarly considered that associating students was ‘one of the hardest parts’ due to the different traditions of student involvement in the HEIs making up the alliance. This led to ‘at least 6 answers from each of the institutions.’ For example, in Charles University and the University of Milan, students were very involved in decision-making. This was not the case at other institutions within the alliance.

The governance structure of EUGLOH featured students most prominently. It was the only alliance that had a student board as part of their governance structure (see Fig. 5). Beyond this dedicated board, a participant also highlighted the intervention of students at all levels of governance. The participant also shared that it was not always easy for students to find their voice in these upper governance layers.

## Cartography and decalcomania: between experimentation and European objectives

A rhizome is a map and the pilot phases of the European Universities Initiative function like a cartography. A participant from the DG EAC stated that the aims of the initiative ‘naturally came from the ground. The room of manoeuvre is quite large. Under this pilot phase (…), we are mapping out the structures that function and we are working to determine what constitutes success. We also leave room to failure’ (DG EAC). The initiative is therefore intended to partly function as an on-going process rather than a project with pre-defined specifications. As part of the 4EU + alliance, the university of Milan saw the need for experimentation establishing that they would ‘experiment with a mobility model based both on in-person and remote attendance for students, instructors, and staff’ (University of Milan, 2020b). Similarly, LMU considers that being part of EUGLOH was an opportunity ‘to experiment with new forms of mobility featured in the next Erasmus programme generation’ (Ludwig Maximilian University of Munich, 2020).

The alliances recognised the novelty of the challenges they faced. According to the CIVICA mission statement, the alliance ‘represents a new challenge to raise the bar in the arena of strategic alliances, being much more pervasive and impactful than any other existing alliance’ (CIVICA, 2019). As part of CIVICA, the Stockholm School of Economics declares that it ‘is determined to work transnationally to find solutions for our mutual challenges’ (Stockholm School of Economics, 2020). The 4EU + mission statement establishes that ‘in order to transform our organisations, we have decided to implement a true university governance for 4EU + rather than a project governance’ (4EU+, 2020). One of the participants from EUGLOH mentioned the challenges they anticipated when confronting contemporary political issues in the EU, such as working with institutions in Viktor Orban’s Hungary. However, the participant compared the overall process to ‘a spinning whirligig’ where ‘things change, and colours change.’ The same interviewee spoke about the responsibility that came with each alliance’s experimental power; they needed to be ‘righteous, pro-active with proposals, models, and pilots.’ The formation of the EUGLOH alliance was described as something ambitious, ‘very heavy and very risky too.’ The stakes were seen as very high, but overall it was considered to be ‘a beautiful bet.’ One programme manager contrasted the European University Initiative with previous ERASMUS + initiatives, defined as ‘projects’ in a traditional sense. He stated that ‘project is the last word I would use to describe the European Universities Initiative. I would call it a process, a very complex process.’

The interviewed staff members of the DG shared that they were ‘testing what would be the vision for the future based on these pilots.’ In other words, these alliances were seen as an experiment rather than a reproduction. Yet, as all interviews were conducted at the start of the initiative and only reflect one point in time, this study cannot confirm the nature of the process of the formation of alliances as experimental. Instead, it would be more appropriate to argue that the study revealed a desire to experiment as part of the formation of university alliances.

Despite experimentation being central to the initiative, the Council of the European Union established that the goal was to rely on ‘the outcomes of the pilot project, to reflect on the future shape of “European Universities”’ (Council of the European Union, 2018). The pilot phases were also there to create tracings from the experimental maps drawn by the alliances. The Council sought to capitalise ‘on the pilot “European Universities” experiences and lessons learnt to inform policy-making and the further development of related cooperation in education and training, and by exploring the need to take forward appropriate policies for the European Universities’ (Council of the European Union, 2019). The Erasmus + programme guide is clear on the fact that although experimentation is encouraged, the alliances should ‘act as models of good practice to progressively further increase the quality, international competitiveness and attractiveness of the European higher education landscape’ (European Commission, 2018b). The University of Milan’s Erasmus policy statement refers to the main objective of the 4EU + pilot phase as ‘to build an advanced University model for education, research and management of student and library services’ (University of Milan, 2020a). Thus, the end goal of the pilot phase was to create tracings for the universities of the future.

## Concluding thoughts

European university alliances are a novel phenomenon that builds on a long-standing idea of creating university networks within Europe. The European Commission presents the European Universities Initiative as paving the way for the future of higher education in the European Union, with its alliances serving as role models for higher education institutions in the EU for years to come. These transnational alliances are to embody the future of higher education in the EU with the ambitious objective to formalise the existence of European Universities by 2025 (European Commission, 2020b). European Universities are still at the start of their endeavour. For the moment, the alliances that have been formed as part of the first pilot phase are non-permanent. Although the European institutions, the alliance coordinators, and participating HEIs hope to pursue this adventure beyond the temporality of the first pilot phases, it is still unclear what is to come. Much remains to be achieved: removing legal and administrative barriers between member states for strengthened cooperation, developing sustainable and long-term funding mechanisms, and building a new governance framework for higher education in the EU.

This study made use of the concept of the rhizome as a conceptual and methodological approach to discuss the formation of alliances in the European Universities Initiative. Drawing on the rhizomatic principles of asignifying rupture, connection, heterogeneity, multiplicity, cartography, and decalcomania, this paper explored the different paths that three university alliances took to achieve common objectives and form novel networks while building on existing ones. The rhizome was intended to be more than a botanical metaphor. This paper used the six principles of the rhizome to compare the heterogeneous networks that make up the first pilot phase of the initiative. Each principle offers a picture of how networks are formed, where they come from, what they are made of, what they are building, and how they move. Future research on these alliances might focus on the principles of multiplicity, cartography, and decalcomania to examine the processes of experimentation within European university alliances. This would require collecting data at different points in time to reflect how the desire to experiment effectively mutates into experimental processes. Rhizomes could establish a cartography of these experimentations to show how the alliances and participating HEIs are trying things out, launching new connections, making wrong turns, meeting dead ends, or celebrating successes.

## Appendix

**Table:**

Name of Institution	Name of Document	
European Council		
	Gottenburg Leaders Agenda Note on Education and Culture	
	Council Conslusions	2017
European Commission		2017
	The Commission’s Contribution to the Leaders’ Working Lunch	2017
	Strengthening European Identity through Education and Culture	2017
	Building a stronger Europe: the role of youth, education and culture policies	2018
	Mapping of European Transnational Collaborative Partnerships in Higher Education	2018
	Call for proposal 2019 Erasmus + Programme	2018
	Erasmus + Programme Guide 2019	2018
	Laying the foundations of the European Education Area	2019
	European Universities Factsheets	2019
	Factsheet CIVICA	
	Factsheet EUGLOH	
	Factsheet 4EU +	
Council of the European Union		
	Draft Council conclusions on moving towards a vision of a European Education Area	2018
	Council Resolution on further developing the European Education Area to support future-oriented education and training systems	2019
4EU +		
	Mission Statement	
	Mobility within 4EU + 6 universities, 3 challenges	
Charles University		
	Startegic Plan 2016–2020	
	Strategy of Internationalisation 2018–2021	
	Erasmus Policy Statement	Date missing
Heidelberg University		
	European Policy Statement 2014–2020	
	European Policy Statement 2021–2027	Date missing
Sorbonne University		
	Projet d'établissement 2019–2023	
Copenhaguen University		
	Erasmus Policy Statement 2014–2020	
	Erasmus Policy Statement 2021–2027	
University of Milan		
	Building an international network	
	Erasmus Policy Statement 2021–2027	
	Strategic Plan 2020–2022	
University of Warsaw		
	Erasmus Policy Statement 2021–2027	
	Erasmus Policy Statement 2014–2020	
CIVICA		
	Mission Statement	
Sciences Po		
	Erasmus Policy Statement 2021–2027	
Bocconi University		
	Erasmus Policy Statement 2014–2020	
	Erasmus Policy Statement 2021–2027	
Central European University		
	Erasmus Policy Statement 2014–2020	
	Erasmus Policy Statement 2021–2027	
European University Institute		
	Erasmus Policy Statement	
	EUI strategy 2019–2024	
Hertie School		
	Erasmus Policy Statement 2014–2020	
	Erasmus Policy Statement 2021–2027	
SNSPA		
	Erasmus Policy Statement 2021–2027	
Stockholm School of Economics		
	Erasmus Policy Statement 2014–2020	
	Erasmus Policy Statement 2021–2027	
EUGLOH		
	Plaquette EUGLOH	
Ludwig Maximilian University of Munich		
	Erasmus Policy Statement 2021–2027	
Lund University		
	Erasmus Policy Statement 2014–2020	
	Erasmus Policy Statement 2021–2027	
Paris Saclay		
	Erasmus Policy Statement 2014–2020	
	Presentation of Erasmus Projects 2014–2020 and 2021–2027	
University of Szeged		
	Erasmus Policy Statement 2014–2020	
	Erasmus Policy Statement 2021–2027	
University of Porto	Erasmus Policy Statement 2014–2020	
	Erasmus Policy Statement 2021–2027	
